# Discovery of inflammatory bowel disease-associated miRNAs using a novel bipartite clustering approach

**DOI:** 10.1186/s12920-020-0660-y

**Published:** 2020-02-24

**Authors:** Md. Altaf-Ul-Amin, Mohammad Bozlul Karim, Pingzhao Hu, Naoaki ONO, Shigehiko Kanaya

**Affiliations:** 1 Nara Institute of Science and Technology, Ikoma 630-0192, Japan; 2 University of Manitoba, Winnipeg, Canada

**Keywords:** IBD, BiClusO, MRM (miRNA regulatory module), MTI (miRNA target interaction)

## Abstract

**Background:**

Multidimensional data mining from an integrated environment of different data sources is frequently performed in computational system biology. The molecular mechanism from the analysis of a complex network of gene-miRNA can aid to diagnosis and treatment of associated diseases.

**Methods:**

In this work, we mainly focus on finding inflammatory bowel disease (IBD) associated microRNAs (miRNAs) by biclustering the miRNA-target interactions aided by known IBD risk genes and their associated miRNAs collected from several sources. We rank different miRNAs by attributing to the dataset size and connectivity of IBD associated genes in the miRNA regulatory modules from biclusters. We search the association of some top-ranking miRNAs to IBD related diseases. We also search the network of discovered miRNAs to different diseases and evaluate the similarity of those diseases to IBD.

**Results:**

According to different literature, our results show the significance of top-ranking miRNA to IBD or related diseases. The ratio analysis supports our ranking method where the top 20 miRNA has approximately tenfold attachment to IBD genes. From disease-associated miRNA network analysis we found that 71% of different diseases attached to those miRNAs show more than 0.75 similarity scores to IBD.

**Conclusion:**

We successfully identify some miRNAs related to IBD where the scoring formula and disease-associated network analysis show the significance of our method. This method can be a promising approach for isolating miRNAs for similar types of diseases.

## Background

Inflammatory bowel disease (IBD) results in disorders in different digestive organ parts with prolonged pain and disruption. The specific causes of IBD, including ulcerative colitis (UC) and Crohn’s disease (CD), remain unknown. Epidemiology of IBD shows the increasing rate by every year where prevention or cure of this disease is still intractable [1]. Even different risk factors, such as ethnicity, smoking, age, family history and gender, are attributed to the IBD, scientists are trying to find other evidence by analyzing the IBD related genomic data. Recently it has been discovered that non-coding RNAs (ncRNAs) are able to control gene expression in a sequence specific manner. Among various types of ncRNAs, MicroRNAs (miRNAs) appear as important cytoplasmic regulators of gene expression. miRNAs are non-coding RNAs of the approximated length of 22 nucleotides, playing important roles in gene splicing and post-transcriptional regulation of gene. Recent studies revealed that there is a strong connection between the regulatory mechanism of miRNA and disease etiology [2–4]. As an example, Overexpression of miR-21 was found in mice to contract pre-B malignant lymphoid-like phenotype tumors. Complete tumor regression is achieved by inactivating this miRNA [5]. Therapeutic inhibition of miRNAs using antisense oligomers (called antimiRs) has also been shown to reduce tumor growth [6]. Scientists have developed miRNA - target interaction (MTIs) databases based on different proven scientific methods which can be used to drill down the functional modules of specific miRNA sets and their target interactions. Previously we developed methods to identify the IBD associated genes from the integrated analysis of transcriptome data and protein-protein interactions (from HIPPIE database). We also compared our results with three different databases namely HuGENet, DisGeNet, CTD and another genome wide association study (GWAS) with respective IBD genes of 849, 866, 129 and 335. Finally we identified a group of IBD related genes with different confidence scores [7]. A miRNA-regulatory module (MRM) is a subset of MTIs where groups of miRNAs participate cooperatively by regulating a bunch of genes to control different biological processes [8]. The MTIs can be represented as a bipartite graph. A bipartite graph is a network of two disjoint sets of nodes where each edge connects a node from one set to a node from the other set. No edge is allowed within any single set. A bicluster is a high density (in terms of connected edges) subgraph of a bipartite graph. There are various applications of biclustering in different fields of study. In biology, gene expression under certain conditions forms a bipartite network which helps to identify the cellular response, disease diagnosis and pathway analysis. Biological network analysis of the pairwise combinations of protein, miRNA, metabolite, conserved functional subsequences, and factor binding sites can predict or understand different cellular mechanisms. Graph convolutional and deep learning methods are also popular technique on prioritizing or predicting the outcome of a gene or disease from such network [9–11]. In the current work, we mainly focused on MRM detection from MTIs by a new biclustering approach we recently developed [12, 13]. We then searched the IBD related genes in MRMs detected in MTI networks. We evaluated the relevance of the miRNAs with IBD by counting their occurrences in different MRMs and their interactions with known IBD genes. Finally, we normalized the score of each miRNA for different MTIs database and evaluated the importance of different miRNA.

## Methods

### IBD gene set

We previously proposed a method for predicting IBD risk genes based on currently known IBD risk genes collected from DisGeNet database and differentially expressed genes determined using gene expression data [7]. In that work we created a disease relevant Protein-Protein Interaction (PPI) network by selecting data from Human Integrated Protein-Protein Interaction reference (HIPPIE) database and then determined high density clusters in the PPI network utilizing DPClusO algorithm [14–16]. Finally, from the statistically significant clusters, we determined 909 genes as potential IBD genes as our novel predictions. We also downloaded IBD related genes from other online sources such as HuGENet [17], Comparative Toxicogenomics Database(CTD) [18], DisGeNet [19] and literatures related to genome wide association study (GWAS) [20–23]. By combining all data, we created a set of IBD related genes comprising 2245 genes.

### miRNA-mRNA/Gene Interaction dataset

miRNAs act as post-transcriptional regulators of the target messenger RNAs (mRNAs) via degradation and/or translational repression. Each miRNA can be linked to a gene. There are databases that have accumulated information of interactions between miRNAs and their target mRNAs/genes. We have collected such information from four different online databases as follows: mirWalk (http://mirwalk.umm.uni-heidelberg.de/) [24], DIANA (http://diana.imis.athena-innovation.gr) [25], miRecords (http://c1.accurascience.com) [26], miRTarbase (http://mirtarbase.mbc.nctu.edu.tw) [27]. Sometimes in a database there are multiple entries of the same miRNA-mRNA interacting pairs which are different in terms of other attributes such as tissue sample, binding probability, binding site position, cell line, tissue, disease category etc. Hence, we used below criteria to select the interactions from these databases. For mirWalk, each mRNA-miRNA interaction has at least 2 evidences (duplicate in the database) and for DIANA, it has at least 4 evidences since more evidences identified for a given mRNA-miRNA show the interaction has higher quality. For miRTarbase, we selected the interaction with the term ’Functional MTI’ since they have higher quality than ’Non-functional MTI’ tagged interactions. Table 1 shows the number of interactions, and associated number of miRNAs and genes we selected for the current study from the four different databases.

### MRM extraction

**Fig. 1:**
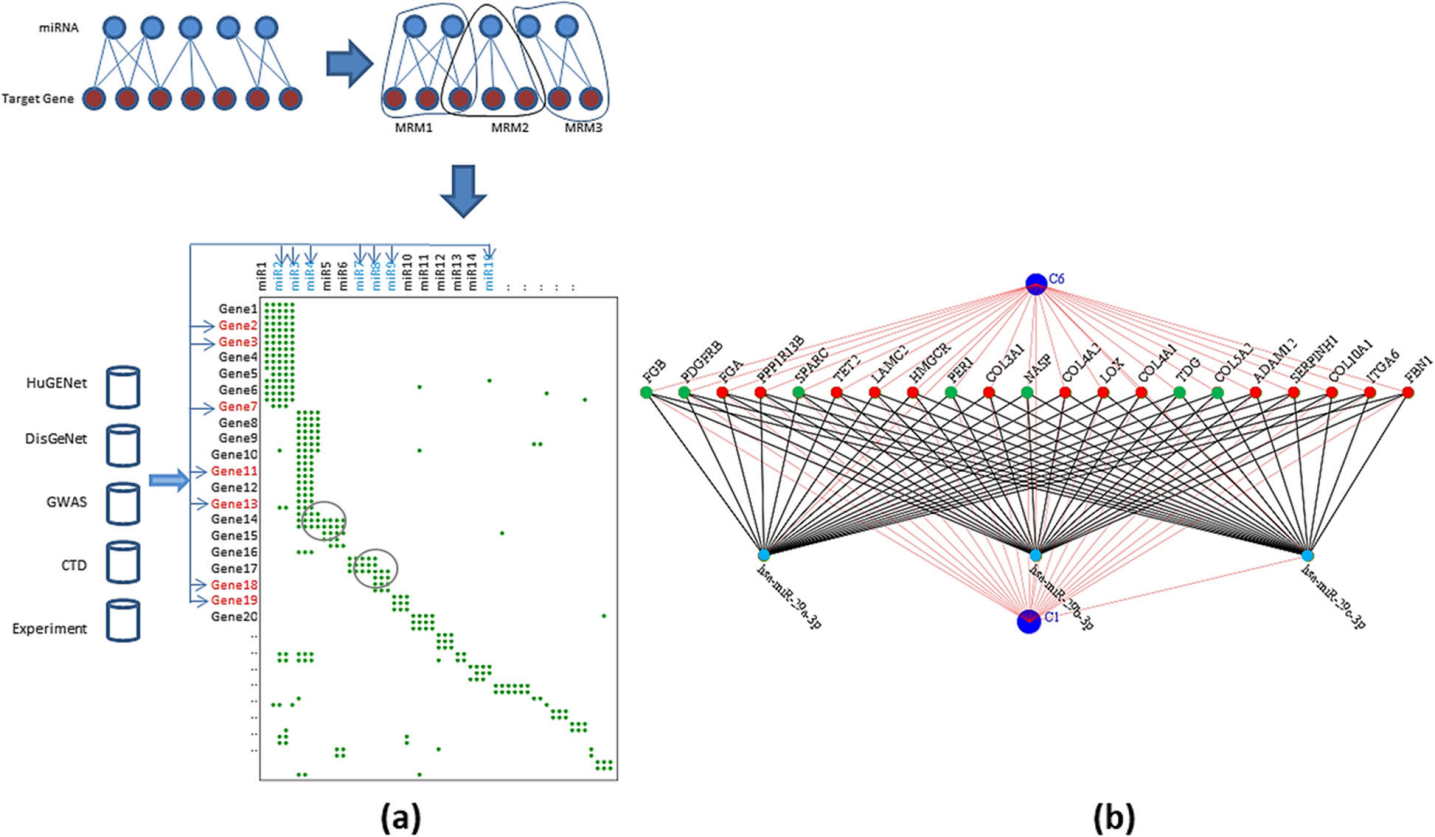
Flow of the proposed approach; **a**) Finding MRMs (upper). Mapping IBD genes in MRMs and finding corresponding sub-MRMs(lower) **b**) A typical sub-MRM from an MRM

The interactions between miRNAs and mRNAs can be represented as a bipartite graph which is called miRNA - target interaction (MTI) network. A bipartite graph is a network of two disjoint sets of nodes where each edge connects a node from one set to a node from the other set. No edge is allowed within any one set. A bicluster is a high density (in terms of connected edges) subgraph of a bipartite graph. In an MTI bipartite network, the miRNAs are a set of nodes and mRNAs are the other set of nodes. The biclusters in an MTI are called miRNA-regulatory modules (MRM). We recently developed a biclustering algorithm called BiClusO [12, 13]. This algorithm was mainly developed for identifying biclusters from a bipartite graph as the miRNA-mRNA network we used in this study. Since a given miRNA can bind to different sets of mRNAs, which implies a given miRNA can be found in different MRMs or biclusters. Based on our algorithm, the bicluster set from a bipartite graph can be overlapped to a certain degree i.e any node may belong to more than one cluster. This is an inherent property of the bipartite graph. The basic theory of BiClusO is to convert a two dimensional problem to one dimensional one by data folding, solve it by one-dimensional algorithm and unfold it again. Thus, the BiClusO algorithm first converts the bipartite graph to a simple graph by taking any node set and measuring the association between those node pairs using relation number and Tanimoto coefficient, then performs simple graph clustering using the polynomial-time heuristic algorithm DPClusO we developed before [14]. Finally, the attachment of the nodes from the second set creates each bicluster. Figure 1a shows the flow of extracting MRMs from a MTI network by BiClusO. Two examples of overlapping between biclusters are indicated by circles in the lower part of Fig. 1a. BiClusO algorithm generates a reasonable number of overlapping biclusters under the optimized parameter settings [7, 12]. In the current work for BiClusO we utilized the following parameter setting: cluster density =0.5, cluster property =0.5, relation number =3, Tanimoto coefficient =0.33 and attachment probability =0.5. Each bicluster is called an MRM. A typical MRM is constructed by a set of miRNAs that are strongly connected with a set of genes. An MRM contains system level information on relations between miRNAs and genes. From the MRMs we created IBD related sub-MRMs by identifying the presence of IBD genes. As mentioned above we selected 2245 IBD genes from different databases and studies. For each bicluster, these genes were matched and corresponding miRNAs were separated. Thus IBD related sub-MRMs were generated. Figure 1b shows a typical sub-MRM from a MRM. The green-colored nodes in the gene side are IBD genes. Red-colored nodes indicate the non IBD genes and aqua colored nodes are the attached miRNA in this MRM. The blue nodes attached by thin red edges are overlapping MRMs to this MRM. Usually, the total number of sub-MRMs is less than the number of MRMs and the size of each sub-MRM is less than the size of the corresponding MRM.

### Relevance Score Calculation

We generated IBD related sub-MRMs from 4 different MTIs (as mentioned in Table 1) separately. Within each set of sub-MRMs, we calculated the relevance score of individual miRNA as a measure of its relation with IBD by using the following formula

1 $$ RS_{miRNA(i)}=NoofIBD_{miRNA(i)} * Noofcluster_{miRNA(i)}  $$

**Table 1:** Number of interactions, miRNA and mRNA on different datasets

Dataset	Interactions	miRNA	Gene
mirWalk	17,290	51	4621
miRTarbase	8157	735	2756
miRecords	1710	249	1097
DIANA	17,535	521	5491

Here

*RS*_*miRNA*(*i*)_= Relevance score of *i*^*th*^ miRNA

*NoofIBD*_*miRNA*(*i*)_= number of IBD genes attached to *i*^*th*^ miRNA in the IBD MRM set

*Noofcluster*_*miRNA*(*i*)_= number of IBD MRMs attached to *i*^*th*^ miRNA

The relevance score signifies the attachment of each miRNA to different sub-MRMs and IBD genes. The total number of interactions, miRNAs, and genes are different among the datasets used in our experiment. We downloaded the latest updated versions of the datasets and observed that the collecting method and importance of each interaction might vary in terms of attributes and parameters in different data sources. Interactions included in more than one dataset might be more accurate and important. Therefore, for a combined ranking of the miRNAs in terms of their relevance to IBD genes is needed. After finding the miRNA sets and their corresponding relevance score from the four different datasets, all sets were merged. We normalized the score of individual miRNA in each dataset and proposed an overall score for each miRNA as follows

2 $$ TRSmiRNA_{i} = \sum_{n=1}^{4}\frac{{RSn_{i}}}{{C_{n}}}\sum_{n=1}^{4}En_{i}  $$

Here

*TRSmiRNA*_*i*_ is the total relevance score of *i*^*th*^ miRNA based on all dataset

*RSn*_*i*_ is the relevance score of *i*^*th*^ miRNA in nth dataset

*Cn*_*i*_ the number of cluster in *n*^*th*^ dataset

*En*_*i*_ is the Boolean value measuring whether *i*^*th*^ miRNA is in the *n*^*th*^ dataset

As an example, hsa-let-7b-5p was found in three datasets: DIANA, mirTarbase, and mirWalk. The total number of biclusters generated from DIANA, mirTarbase, and mirWalk datasets are 650, 64 and 1579. In DIANA hsa-let-7b-5p was attached to 44 biclusters with 48 IBD genes. In mirTarbase it was attached with 1 bicluster with 1 IBD gene and in mirWalk, it was attached with 209 biclusters with 69 IBD genes. So the relevance score for this miRNA in three datasets are 2112, 1, and 14421. The total score is (2112/650+1/64+14421/1579)(1+1+1) = 37.21

## Results and discussion

miRNA isolating is frequently used in the diagnosis and monitoring of different diseases. Numerous studies have identified miRNAs as a potential biomarker for different diseases. Different databases of miRNA and mRNA interaction are created by compiling experimental results of different studies. Most of the databases have varied attributes with different quality along with miRNA and mRNA. Researchers are updating their databases by collecting the biological and medicinal experimental results. One of the biggest challenges in this work was to select the valid interactions which have strong evidence on the basis of other attribute values. Important attributes and frequency of reported interactions were considered in the selection process and duplicate or triplicate miRNA-mRNA pairs were removed from the final dataset utilized in this study.

### Ranking of the miRNAs

Based on miRWalk dataset, we generated 1579 biclusters from which we found 1011 sub-MRMs encompassing 50 miRNAs and 333 genes. Top 10 miRNAs according to relevance score are hsa-let-7d-5p, hsa-let-7a-5p, hsa-let-7e-5p, hsa-let-7c-5p, hsa-let-7b-5p, hsa-miR-106a-5p, hsa-miR-106b-5p, hsa-let-7f-5p, hsa-let-7i-5p.

64 biclusters were generated from mirTarbase dataset out of which we found 41 IBD related sub-MRMs encompassing 100 miRNAs and 128 genes. Top 10 miRNAs are hsa-miR-221-3p, hsa-miR-29b-3p, hsa-miR-222-3p, hsa-miR-34c-5p, hsa-miR-200c-3p, hsa-miR-29c-3p, hsa-miR-200b-3p, hsa-miR-29a-3p, hsa-miR-34b-3p, hsa-miR-24-3p.

23 biclusters were generated from small dataset miRecords where 20 sub-MRMs with 48 miRNAs and 54 genes were found. Out of them, top 10 miRNAs are hsa-miR-16, hsa-miR-15a, hsa-miR-17, hsa-miR-29a, hsa-miR-181a, hsa-miR-29b, hsa-miR-1, hsa-miR-221, hsa-miR-20a, hsa-miR-34b.

**Fig. 2:**
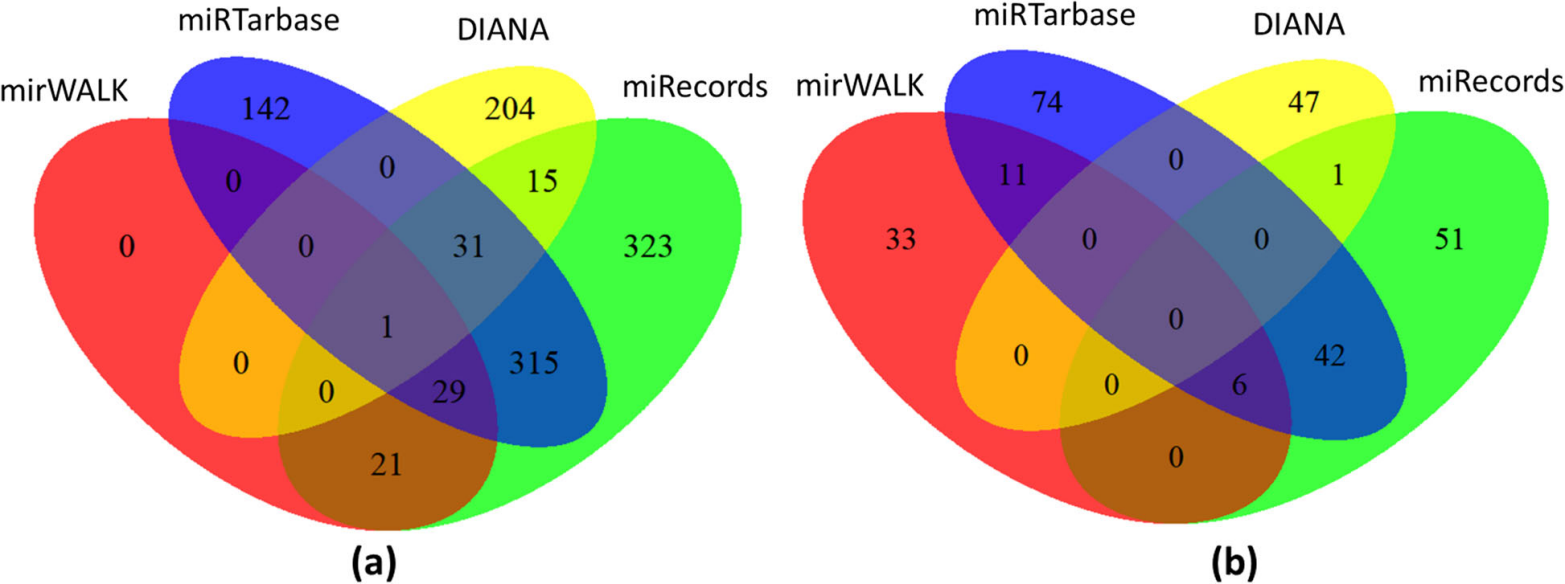
Number of miRNAs in different dataset **a**) before biclustering **b**) After biclustering

**Fig. 3:**
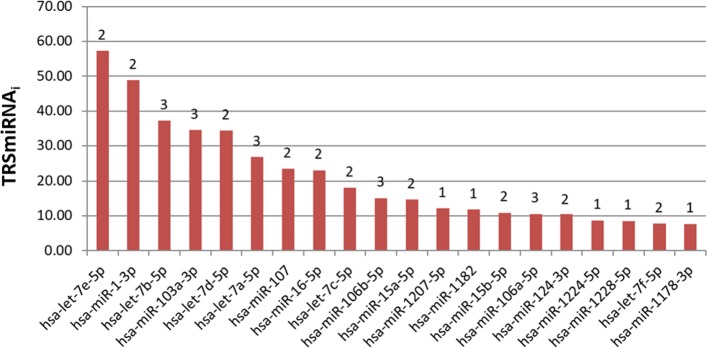
Total score of top 20 miRNAs with number of attachment to different datasets

DIANA dataset produced 650 biclusters with 423 sub-MRMs where 133 miRNAs and 340 genes were found. Top 10 miRNAs in this dataset are hsa-miR-1-3p, hsa-miR-16-5p, hsa-miR-15a-5p, hsa-miR-15b-5p, hsa-miR-124-3p, hsa-miR-103a-3p, hsa-miR-27a-3p, hsa-miR-107, hsa-miR-20a-5p, hsa-let-7b-5p. Venn diagram from Fig. 2 shows the number of miRNAs in different datasets before biclustering (a) and after detection of sub-MRMs (b). From the Venn diagram of Fig. 2a, it is evident that most of the datasets have an almost distinct set of miRNAs. The 265 miRNAs included in Fig. 2b are IBD related miRNAs detected by our approach. Out of them 6 are common in three different datasets and 53 are common miRNAs on two different datasets. Figure 3 shows the top 20 miRNA according to the total relevance score where 15 of them were found in at least two datasets.

### Relevance of the top miRNAs to IBD

We also evaluated the ratio of IBD related genes associated with the top 20 selected miRNAs to those of all of the 265 selected miRNAs and it appears that IBD related genes are enriched in the top 20 miRNAs. The total number of IBD genes attached to the top 20 miRNAs is 493 whereas total number of IBD genes attached to all 265 miRNAs is 664. Thus an approximate ratio of 10:1 is achieved in terms of attachment to the IBD genes for the top 20 miRNAs.

Out of the top 20 selected miRNAs, 6 miRNAs are related to the miR-7 family. miR-7 family has 9 members which are let-7a, let-7b, let-7c, let-7d, let-7e, let-7f, let-7g, let-7i, and miR-98. miRNAs related to this family regulate various biological functions such as cell proliferation, cell cycle, stem cell biology, metabolism, and migration, progression, and chemoresistance. miR-7 is downregulated on different types of cancer such as colon cancer [28], gastric tumors [29] etc. Patients with inflammatory bowel disease (IBD) are at significantly increased risk of colorectal cancer (CRC) [30–32], principally resulting from the pro-neoplastic effects of chronic intestinal inflammation [33].

Using imperfect base pairing to the 3’-UTR the mature let-7 negatively regulates the expression of target mRNAs at a posttranslational level [34]. The expression levels of let-7 microRNAs in stem and progenitor cells are maintained low during the normal development process. Expression levels increase when the progenitor cells differentiate [35]. The downregulation of let-7 promotes migration and invasion of normal intestinal epithelial cells and CRC cells [36]. The downregulation of let-7 or upregulation of either LIN28A or LIN28B has been reported to be related to the prognosis in CRC patients in critical stage. The expression level of LIN28B was inversely correlated to that of mature let-7a in human CRC [36]. From an experiment, 38% out of 600 CRC patients were found to be highly expressed of LIN28A or LIN28B [37]. Let-7 microRNAs are also downregulated in different types of cancers such as hepatocellular carcinoma (HCC), gastric adenocarcinoma, pancreatic cancer, ovarian cancer, prostate cancer, Burkitt lymphoma, renal cell carcinoma, breast cancer, and melanoma [38].

Expression of hsa-let-7e-5p is markedly upregulated in HHM RC. Subsequent assessment of the expression of hsa-let-7e-5p target genes implicated that it may be a prognostic biomarker for RC with HHM [39]. Both inflamed and non-inflamed terminal ileal mucosa in adult patients with active CD have their distinct miRNA expression patterns compared with healthy controls for hsa-let-7b-5p [40]. let-7d has a significant impact on epithelial-to-mesenchymal transition (EMT) and formation of cancer initiating cells which are resistant to irradiation and chemical exposure and responsible for cancer metastasis [41]. In patients with stage II CRC hsa-miR-103a-3p is reported as a promising predictive biomarkers for tumor recurrence [42]. Expression of miR-16 is elevated in CD and UC peripheral blood [43]. Overexpression of miR-106b-5p suppress the CRC cell migration and inhibits the invasion and metastasis of colorectal cancer by targeting CTSA [44].

Upregulation of miR-15a-5p in IBD patient is reported in [45]. By sponging miR-1207-5p a long noncoding RNA BC032469 upregulates hTERT expression which promotes proliferation in gastric cancer [46]. There was a significant negative correlation between miR-1182 and hTERT which attenuates gastric cancer. miR-15b-5p is down-regulated in CRC cells and tissues. The inhibitory effects of miR-15b-5p on cell apoptosis and enhancement of drug sensitivity are mediated by the down-regulation of its NF- *κ*B1 and IKK- *α* targets [47]. Long non-coding RNA FER1L4 exerts tumor suppressive effects on colon cancer by mediating miR-106a-5p repression [48]. The expression level of miR-106a is elevated in Intestinal biopsy, peripheral blood/serum cell of UC and CD patient [49]. mir-124 is downregulated by regulating STAT3 expression in colon tissues of pediatric patients with UC [50]. The expression level of miR-124-3p is increased in the advanced stage of CRC patients. miR-124-3p works as a tumor suppressor gene in astrocytomas by targeting the repression of protein PIM1 [51]. miR-1224-5p has the colitogenic ability in the gut epithelium and is directly associated with IBD disease [52]. miR-1228 is downregulated in gastric cancer tissues also overexpression of mir-1228 significantly inhibited the proliferation and colony formation of gastric cancer cells [53].

### miRNA disease network

**Fig. 4:**
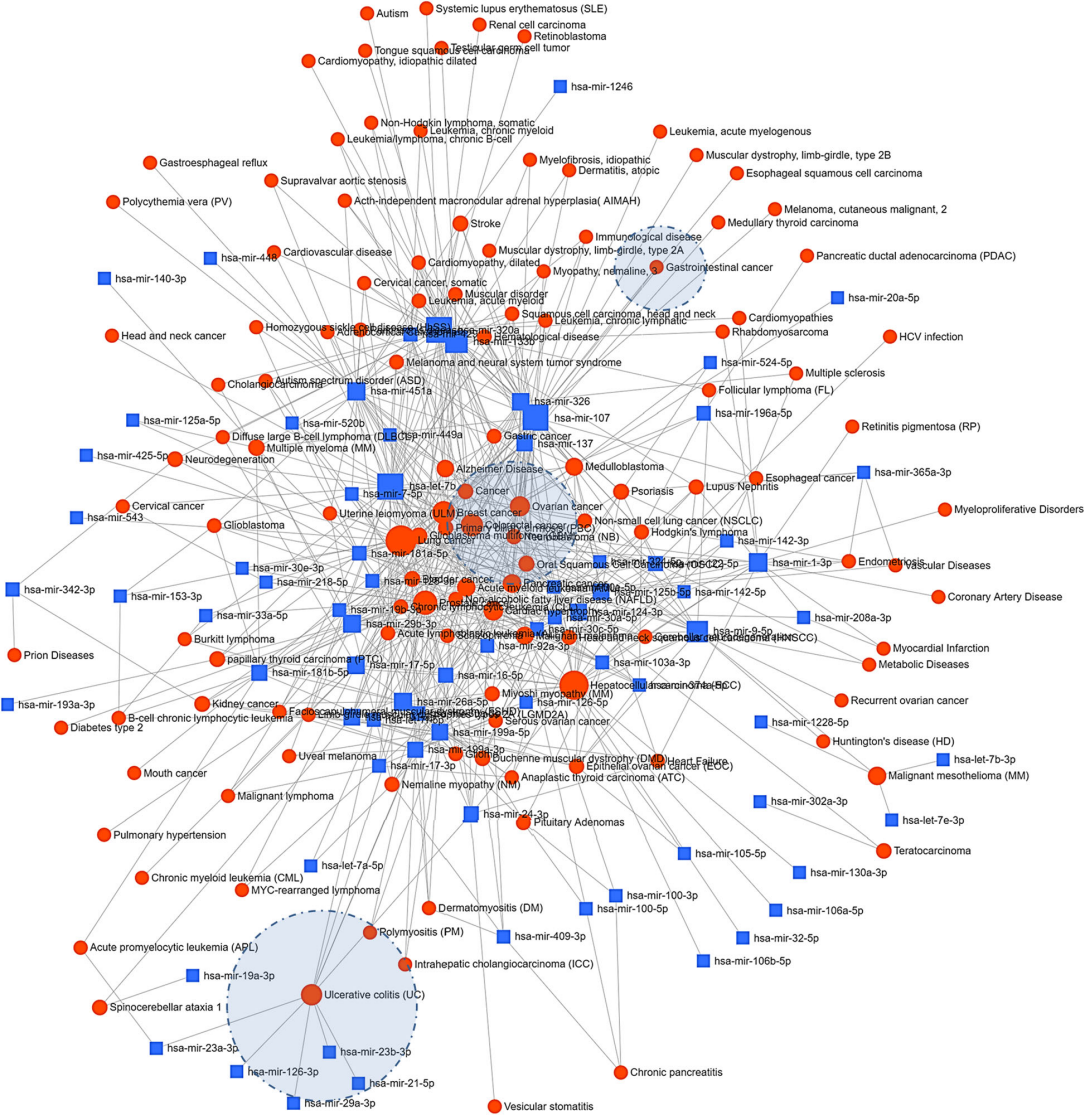
miRNA and Disease network

We used 265 our identified miRNAs to miRNet (https://www.mirnet.ca) and got the associated miRNA-disease network [54]. 74 out of the 265 miRNAs are included in this unweighted network with a minimum degree cutoff = 1. The network is shown in Fig. 4 where the circular nodes correspond to diseases and the rectangular nodes are miRNAs. The sizes of the nodes are proportional to their respective degrees. The layout of Fig. 4 expresses the centralization of the nodes with higher degree hence the association of a disease with a significant number of miRNAs are plotted in the center.

The dotted circles are the highlighted region of three IBD associated diseases i.e. Ulcerative colitis, CRC and Gastrointestinal Cancer. From the network, most of the associations are with different type of cancer e.g Lung cancer, hepatocellular carcinoma (HCC), prostate cancer, breast cancer, colorectal cancer, ovarian cancer, and pancreatic cancer. We also identified ulcerative colitis with 10 different miRNAs and gastrointestinal cancer with two different miRNAs. We found 126 different types of diseases on this network where 20% of them are associated with inflammation. Some of these diseases are Acute lymphoblastic leukemia (ALL), Chronic pancreatitis, Dermatitis, atopic, Dermatomyositis (DM), Multiple sclerosis, Polymyositis (PM), Psoriasis, etc. This also implies that inflammatory pathway analysis related to those diseases can be applied for IBD where inflammation is a common symptom.

### Disease Similarity

**Fig. 5:**
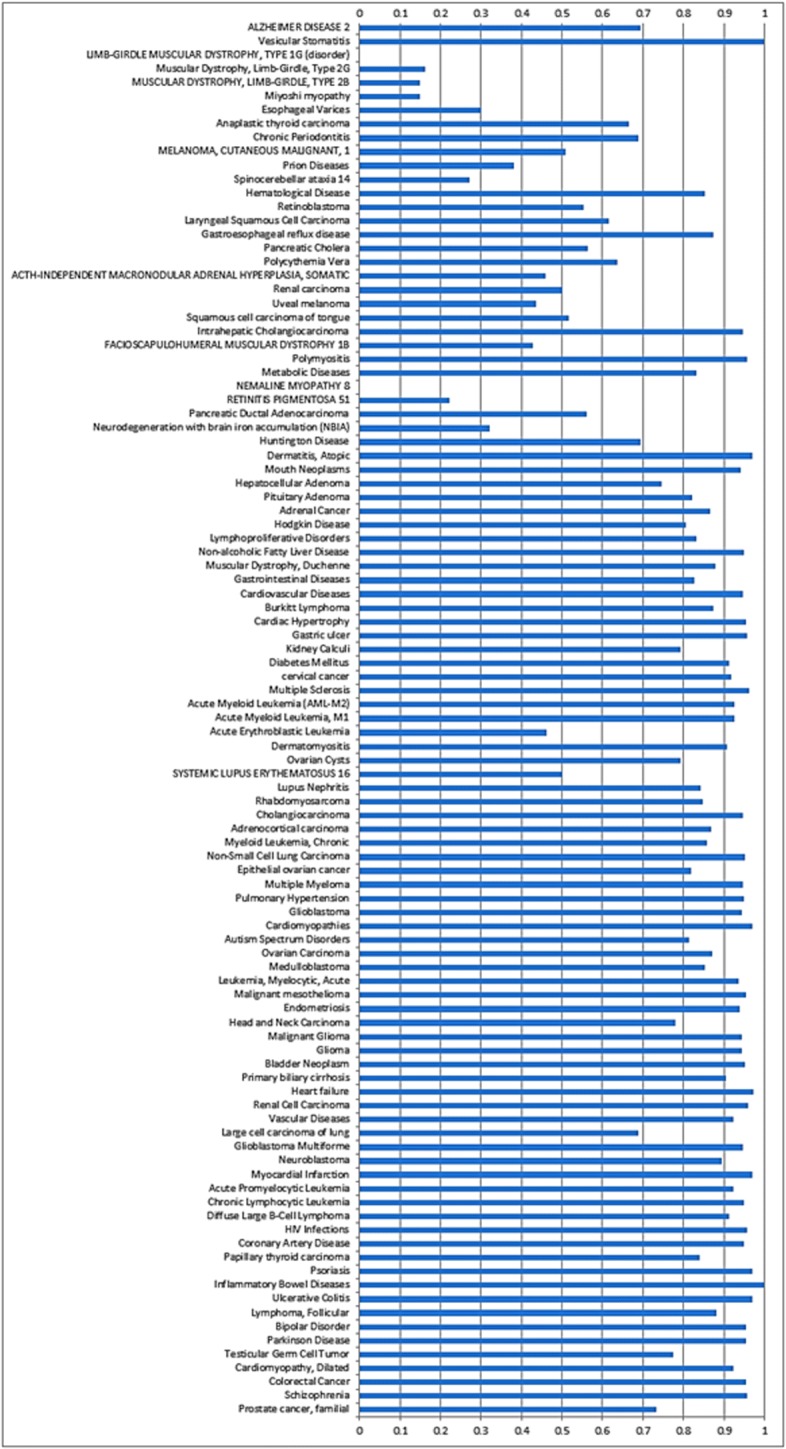
Disease similarity between IBD and different diseases

We also evaluated the similarity of identified disease set from the miRNA-disease network to Inflammatory bowel disease. We used DisGeNET to find those disease and their corresponding gene set. The database contains gene-disease associations from UNIPROT, CGI, ClinGen, Genomics England, CTD (human subset), PsyGeNET, and Orphanet. We used ’DOSE’ package of R and evaluated the disease similarity by using clustersim function. Figure 5 shows the similarity of identified disease set with the Inflammatory bowel disease. Matching score 1 indicates maximum similarity. The results show that 71% diseases are similar to IBD with a score more than 0.75. Therefore, it can be concluded that our approach is a promising method for prioritizing IBD related miRNAs and this method can be applied to other diseases.

## Conclusion

Dysregulation of single or multiple miRNAs can affect normal cellular function i.e proliferation, metabolism, apoptosis, cell cycle, stem cell division, neuronal gene expression which are the major cause of different diseases in human. In recent years scientists have proved the regulation of miRNAs to turn the cancer cell as malignant. Rapid discovery and progress of different clinical experiment accumulate the MRM data that can aid to discover the molecular mechanism of disease development. Our present goal was to narrow down the large domain of the multidimensional database and discover effective information. In this work, we successfully identified some important IBD related miRNAs. We also searched the literature for the association of these miRNAs to UC, CD and similar types of disease like CRC and Gastrointestinal cancer and provided evidences in support of our predictions.

## Data Availability

Biclustering tool BiClusO is available in the link “http://www.knapsackfamily.com/BiClusO/. Data is available on request from the corresponding author.

## Abbreviations

ALL: Acute lymphoblastic leukemia
BiClusO: Biclustering algorithm with overlapping
CD: Crohn’s disease CD
CRC: Colorectal cancer
CTD: Comparative toxicogenomics database
DM: Dermatomyositis
DIANA: A database of miRNA:gene interactions
DisGeNet: Disease gene network
DPClusO: Simple cluster algorithm with overlapping
GWAS: genome wide association study
HCC: hepatocellular carcinoma
HIPPIE: Human integrated protein-protein interaction reference
HuGENet: The human genome epidemiology network
IBD: Inflammatory bowel disease
MRM: miRNA regulatory module
mRNA: Messenger RNA
miRNA: micro RNA
miRecords: A resource for animal miRNA-target interactions
miRTarBase: A curated database of microRNA-target interactions
mirWalk: A comprehensive atlas of predicted and validated miRNA-target interactions
MTI: miRNA target interaction
ncRNA: Non-coding RNA
PM: Polymyositis
PPI: Protein-protein interaction
RS: Relevance score
TRS: Total relevance score
UC: Ulcerative colitis
